# Impact of Lymph Node Involvement on Prognosis and Survival in Patients With Ovarian Carcinoma: A Clinicopathological Correlation Study

**DOI:** 10.7759/cureus.100115

**Published:** 2025-12-26

**Authors:** Zarina Naz, Jaweria Pervaiz, Junaid Azmat, Muhammad Khurrum Islam, Hira Zulfiqar, Nosheen Wahab Salman, Abdul Ahad Mehboob

**Affiliations:** 1 Department of Clinical Academic Management, National University of Medical Sciences, Rawalpindi, PAK; 2 Pathology, Shalamar Hospital, Lahore, PAK; 3 Pathology, Sialkot Medical College, Sialkot, PAK; 4 Medical Oncology, Sargodha Medical College, Sargodha, PAK; 5 Pathology, Shalamar Medical and Dental College, Lahore, PAK; 6 Obstetrics and Gynaecology, Al-Aleem Medical College, Gulab Devi Teaching Hospital, Lahore, PAK; 7 Department of Pathology, National Laboratory and Testing Centre, Lahore, PAK

**Keywords:** lymph node metastasis, ovarian cancers, ovarian carcinoma, prognosis, survival analysis, survival time

## Abstract

Background: Ovarian carcinoma remains a leading cause of gynecologic cancer mortality worldwide, primarily due to its late presentation and high recurrence rates.

Objective: This study aims to determine the impact of lymph node involvement on prognosis and survival in patients with epithelial ovarian carcinoma and to correlate nodal status with clinicopathological parameters.

Methods: This retrospective cross-sectional study was conducted at Shalamar Medical and Dental College, Lahore, from September 2024 to May 2025. A total of 125 patients with histologically confirmed epithelial ovarian carcinoma who underwent primary cytoreductive surgery with lymphadenectomy were included. Data regarding patient demographics, tumor grade, FIGO stage, histologic subtype, and lymph node status were collected.

Results: Lymph node metastasis was identified in 48 patients (38.4%). Node positivity showed a significant association with high-grade tumors (p = 0.02) and advanced FIGO stage (p < 0.001). The mean number of lymph nodes dissected was 14.2 ± 6.8, while the mean lymph node ratio was 0.21 ± 0.14. Patients with lymph node involvement demonstrated a markedly lower three-year overall survival rate of 42.7%, compared with 78.1% in node-negative cases (p = 0.003, log-rank test). On multivariate Cox regression, lymph node positivity (heart rate (HR) = 2.41, 95% confidence interval (CI): 1.38-4.21, p = 0.002) and advanced FIGO stage (HR = 3.05, 95% CI: 1.76-5.18, p < 0.001) emerged as independent predictors of poor survival.

Conclusion: It is concluded that lymph node involvement is a significant independent prognostic factor in epithelial ovarian carcinoma, strongly associated with advanced stage, high tumor grade, and reduced survival. Accurate lymph node assessment should be routinely incorporated into surgical and pathological evaluation for precise staging and individualized management.

## Introduction

Ovarian carcinoma is a major global health concern and remains one of the most lethal malignancies affecting women worldwide. It is the eighth most common cancer among females and the fifth leading cause of cancer-related deaths, accounting for nearly 295,000 new cases and over 185,000 deaths annually, according to GLOBOCAN 2022 [1]. Despite significant advances in diagnostic imaging, molecular genetics, and chemotherapeutic regimens, ovarian cancer continues to carry a poor prognosis due to its insidious onset and frequent late-stage presentation [2]. The majority of women are diagnosed with the disease after it has already spread beyond the ovaries, frequently to the peritoneum and regional lymph nodes, limiting treatment options [3]. The biological behaviour of ovarian carcinoma is complex, influenced by histological subtype, tumor grade, stage, and molecular profile. Among these, lymph node involvement has received a lot of attention as a crucial factor in determining prognosis and treatment plan [4]. The lymphatic system represents one of the earliest and most common routes of metastatic spread in epithelial ovarian cancers. Pelvic and para-aortic lymph nodes serve as regional basins for tumor dissemination, and their status provides essential information for accurate staging according to the FIGO (International Federation of Gynecology and Obstetrics) classification [5]. FIGO stage IIIc, for example, includes metastasis to regional lymph nodes, while distant lymph node metastasis (such as supraclavicular or inguinal) is classified as stage IVb disease [6]. Thus, lymph node assessment not only refines staging accuracy but also significantly influences postoperative treatment planning and patient counseling. The prognostic impact of nodal involvement, however, remains a subject of ongoing debate [7]. Some studies have demonstrated that patients with nodal metastases experience worse progression-free and overall survival compared to those with node-negative disease. By contrast, other research suggests that lymph node-positive patients without extensive peritoneal spread may still achieve relatively favorable outcomes, especially when complete surgical debulking is achieved. This heterogeneity highlights the need for clinicopathological correlation studies to clarify how nodal status affects long-term survival in different disease contexts [8].

 Moreover, the extent of nodal disease appears to matter. The lymph node ratio and the number of nodes retrieved have emerged as potential prognostic factors. While extensive nodal dissection that results in higher retrieval counts has been linked to improved staging accuracy and, in some instances, better outcomes, a higher lymph node ratio is generally associated with poor survival [9]. However, extensive lymphadenectomy is not without risk; it can increase operative time, blood loss, and postoperative complications such as lymphoceles, infection, or lower-limb lymphedema. Hence, determining which patients truly benefit from comprehensive nodal dissection is a critical question for modern gynecologic oncology [10]. From a histopathological perspective, certain tumor characteristics correlate with a higher likelihood of nodal metastasis. The most prevalent histologic subtype, high-grade serous carcinoma, typically presents with extensive nodal involvement and early lymphovascular invasion. On the other hand, clear-cell and mucinous variants spread in different ways and are typically restricted to the surfaces of the ovary or peritoneum [11]. Tumor size, capsular invasion, and the presence of ascites have also been shown to influence the probability of lymphatic dissemination. Predicting which patients are most at risk and might benefit from surgical staging that is more aggressive requires an understanding of these associations. Prognostic stratification and survival prediction are also profoundly affected by the status of lymph nodes [12]. Even after adjusting for age, tumor grade, and stage, studies using large population databases like Surveillance, Epidemiology, and End Results (SEER) have shown that nodal involvement alone predicts lower overall survival [13]. By contrast, some authors have noted a paradoxical survival advantage in patients with isolated lymph node metastases compared to those with peritoneal carcinomatosis, leading to speculation that nodal disease alone might represent a less aggressive biological phenotype. These contradictory findings highlight the need for additional research, particularly from resource-constrained settings where differences in outcomes may be influenced by differences in surgical expertise, access to pathology services, and chemotherapy protocols [14].

Objective

This study aimed to evaluate the impact of lymph node involvement on overall survival (OS) and disease-free survival (DFS) in patients with ovarian carcinoma.

## Materials and methods

This retrospective cross-sectional study was conducted at Shalamar Medical and Dental College, Lahore, from September 2024 to May 2025. A total of 125 patients diagnosed with ovarian carcinoma who underwent primary cytoreductive surgery with lymphadenectomy were included in this study. Non-probability consecutive sampling was used to select eligible cases from hospital pathology archives. The study included all patients with histologically confirmed primary epithelial ovarian carcinoma who underwent either comprehensive surgical staging or cytoreductive surgery with lymph node dissection. Only cases with complete clinical, histopathological, and follow-up data were eligible for analysis. Patients were excluded if they had non-epithelial ovarian malignancies, such as germ cell tumors or sex-cord stromal tumors, or if they had incomplete clinical records or insufficient lymph node sampling.

Data collection procedure

**Figure 1:**
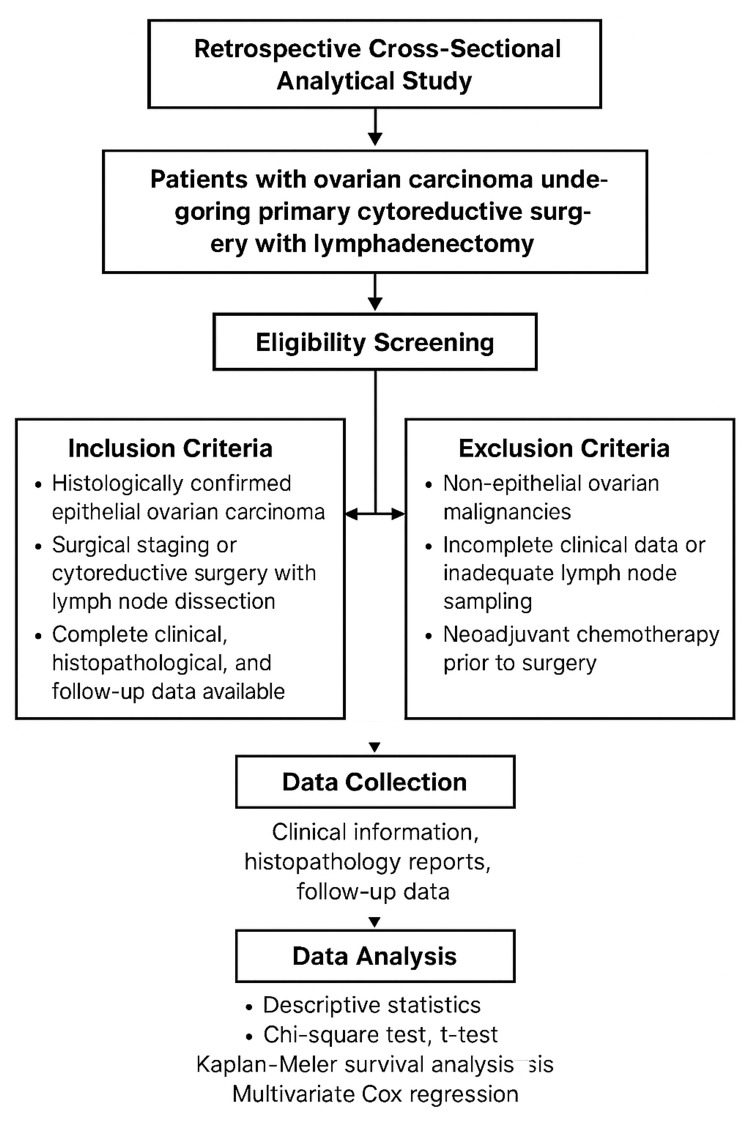
Structural workflow

Patient records were retrieved from the institutional cancer registry and pathology archives. Clinical information, including age, menopausal status, presenting symptoms, and serum CA-125 levels, was obtained from hospital records. Surgical notes were reviewed to determine the extent of disease and the number and location of lymph nodes removed (pelvic and/or para-aortic). Histopathology reports were used to document tumor type, grade, and stage according to the 2021 FIGO classification. Lymph node involvement was recorded as positive or negative based on microscopic evidence of metastatic deposits. The lymph node ratio was calculated by determining the ratio of the number of positive nodes to the total number of nodes isolated. Follow-up data were obtained from medical records and oncology clinic databases to assess OS and DFS. OS was defined as the time from initial surgery to death or last follow-up, and DFS was the time to recurrence or metastasis (Figure 1). 

Data analysis

Data were entered and analyzed using IBM SPSS Statistics for Windows, Version 26.0 (released 2018, IBM Corp., Armonk, NY). Quantitative variables such as age and number of lymph nodes were presented as mean ± standard deviation, while categorical variables such as tumor grade, stage, and nodal status were expressed as frequencies and percentages. The Chi-square test was used to compare categorical variables between lymph node-positive and lymph node-negative groups. Independent t-tests were applied for continuous variables. Kaplan-Meier survival curves were constructed for OS and DFS, and comparisons between groups were made using the log-rank test.

## Results

A total of 125 patients diagnosed with epithelial ovarian carcinoma were included in the study. The mean age of the patients was 54.7 ± 10.8 years, with most being postmenopausal (71; 56.8%) and premenopausal (54; 43.2%). The predominant presenting symptom was abdominal distension (75; 60%), followed by pelvic pain (58; 46.4%) and weight loss (27; 21.6%). Elevated serum CA-125 levels (>35 U/mL) were observed in 102 patients (81.6%). The majority of participants were from urban areas (83; 66.4%) (Table 1).

**Table 1:** Baseline demographic and clinical characteristics (N = 125)

Variable	Category	n (%) or mean ± SD
Age (years)		54.7 ± 10.8
Menopausal status	Premenopausal	54 (43.2)
	Postmenopausal	71 (56.8)
Presenting symptom	Abdominal distension	75 (60.0)
	Pelvic pain	58 (46.4)
	Weight loss	27 (21.6)
Serum CA-125 > 35 U/mL		102 (81.6)
Residence	Urban	83 (66.4)
	Rural	42 (33.6)

Histopathological evaluation revealed that high-grade serous carcinoma was the most common subtype, found in 86 patients (68.8%), followed by endometrioid carcinoma (20; 16%), mucinous carcinoma (11; 8.8%), and clear-cell carcinoma (8; 6.4%). Tumor grading showed that 59 (47.2%) tumors were poorly differentiated (Grade III), while 48 (38.4%) were moderately differentiated (Grade II), and 18 (14.4%) were well differentiated (Grade I). Based on FIGO 2021 staging, Stage III disease predominated 57 (45.6%), followed by Stage II (36; 28.8%), Stage I (20; 16%), and Stage IV (12; 9.6%) (Table 2).

**Table 2:** Histopathological and stage distribution

Parameter	Category	n (%)
Histologic subtype	High-grade serous carcinoma	86 (68.8)
	Endometrioid carcinoma	20 (16.0)
	Mucinous carcinoma	11 (8.8)
	Clear-cell carcinoma	8 (6.4)
Tumor grade	Grade I – Well differentiated	18 (14.4)
	Grade II – Moderately differentiated	48 (38.4)
	Grade III – Poorly differentiated	59 (47.2)
FIGO stage (2021)	I	20 (16.0)
	II	36 (28.8)
	III	57 (45.6)
	IV	12 (9.6)

Lymph node metastasis was identified in 48 patients (38.4%). Among these, pelvic node involvement was most frequent (29; 60.4%), followed by para-aortic nodes (13; 27.1%) and both regions (6; 12.5%). The mean number of lymph nodes dissected was 14.2 ± 6.8, with an average of 3.1 ± 2.4 positive nodes, yielding a mean lymph node ratio (LNR) of 0.21 ± 0.14. Lymph node positivity correlated significantly with high tumor grade (p = 0.02) and advanced FIGO stage (p < 0.001), but not with age (p = 0.38). The mean serum CA-125 level was substantially higher in node-positive patients (480.3 ± 120.7 U/mL) than in node-negative ones (297.5 ± 92.6 U/mL, p = 0.01) (Table 3).

**Table 3:** Distribution and clinicopathological correlation of lymph node involvement

Variable	LN-Positive (n = 48)	LN-Negative (n = 77)	p-value
Mean age (years)	55.8 ± 11.1	53.9 ± 10.4	0.38
High-grade tumor	31 (64.6)	28 (36.4)	0.02 *
Stage III–IV disease	42 (87.5)	27 (35.1)	<0.001 *
Mean CA-125 (U/mL)	480.3 ± 120.7	297.5 ± 92.6	0.01 *
Pelvic node positive	29 (60.4)	—	—
Para-aortic node positive	13 (27.1)	—	—
Both regions positive	6 (12.5)	—	—
Mean nodes dissected	14.2 ± 6.8	—	—
Mean positive nodes	3.1 ± 2.4	—	—
Mean lymph node ratio (LNR)	0.21 ± 0.14	—	—

**Figure 2:**
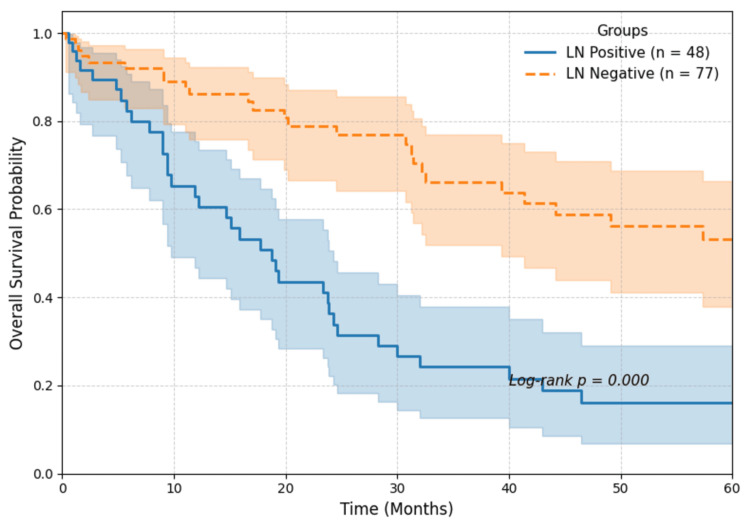
Kaplan–Meier survival curve by lymph node status

Multivariate Cox regression analysis identified lymph node positivity as an independent predictor of poor OS (HR = 2.41; 95% CI: 1.38-4.21; p = 0.002). Similarly, advanced FIGO Stages III-IV significantly increased mortality risk (HR = 3.05; 95% CI: 1.76-5.18; p < 0.001), while high tumor grade also adversely affected survival (HR = 1.79; 95% CI: 1.01-3.11; p = 0.04). Age > 60 years was not a significant predictor (p = 0.46) (Table 4, Figure 2).

**Table 4:** Multivariate Cox regression analysis for overall survival

Variables	Hazard ratio (HR)	95% confidence interval (CI)	p-value
Lymph node positivity	2.41	1.38–4.21	0.002*
FIGO Stage III–IV	3.05	1.76–5.18	<0.001*
High-grade tumor	1.79	1.01–3.11	0.04*
Age > 60 years	1.22	0.71–2.09	0.46
Exploratory: Pelvic + para-aortic LN involvement vs isolated pelvic LN involvement	1.62	0.92–2.84	0.08 (ns)

## Discussion

Due to its late clinical presentation and high recurrence rates, ovarian carcinoma remains one of the deadliest gynecologic cancers worldwide. Despite advances in surgical and chemotherapeutic strategies, survival outcomes for many patients remain suboptimal. In this context, the prognostic value of lymph node involvement has been a subject of continuous debate. Lymph node metastasis and clinical outcomes, such as OS and DFS, were correlated with histopathological and clinical parameters in the current study, which included 125 patients with histologically confirmed ovarian carcinoma. Our study demonstrated that lymph node metastasis occurred in approximately 38.4% of patients, with a higher incidence among those presenting at advanced FIGO stages (III-IV) and with high-grade serous carcinoma [12]. Lymph node involvement was found to correlate significantly with poor differentiation, elevated CA-125 levels, and reduced OS and DFS. Even after adjusting for tumor grade and FIGO stage, multivariate Cox regression confirmed that lymph node positivity was an independent predictor of a worse prognosis. These results reinforce the concept that nodal status serves as a robust marker of tumor aggressiveness and metastatic potential in ovarian carcinoma [13]. When stratified by histologic subtype, our findings revealed a predominance of high-grade serous carcinoma (68.8%), consistent with previous reports where serous histology accounts for over two-thirds of advanced-stage ovarian cancers. This subtype's predilection for lymphovascular invasion and widespread peritoneal dissemination explains its frequent association with nodal metastasis. A notable observation in our study was the significant association between advanced FIGO stage and nodal involvement (p < 0.001). Lymph node metastasis was nearly three times more likely in patients with stage III-IV disease than in those with early-stage tumors. This relationship is expected, as tumor burden and serosal infiltration facilitate lymphatic permeation [14].

This backs up the National Comprehensive Cancer Network (NCCN) and European Society for Medical Oncology & European Society of Gynaecological Oncology (ESGO-ESMO) consensus guidelines that call for selective lymphadenectomy even in cases of ovarian cancer that appear to be in its early stages to ensure accurate staging and the best possible planning of adjuvant therapy. Our findings also support the prognostic significance of the lymph node ratio (LNR), defined as the number of positive nodes divided by total nodes removed. In addition, the influence of preoperative systemic treatment cannot be overlooked, as neoadjuvant chemotherapy may modify lymph node burden and alter survival patterns by downstaging disease before surgery, potentially confounding the true prognostic impact of nodal involvement. Several studies have shown that higher LNR values independently predict poorer survival outcomes [15]. Patients with LNRs greater than 0.2 outperformed those with lower ratios in terms of three-year OS and DFS, indicating that LNRs may be a more accurate indicator of metastatic burden than absolute node count. Lymph node involvement is traditionally regarded as a hallmark of advanced disease. However, its precise prognostic value varies among studies. When complete cytoreduction is achieved, some previous studies suggested that nodal metastasis does not independently worsen outcomes. Our results, in contrast, indicate that even after accounting for stage and grade, nodal positivity independently reduced OS (HR = 2.41, p = 0.002) [16]. Another important consideration is the heterogeneity of lymph node burden, as current staging systems classify patients with a single positive lymph node and those with extensive locoregional nodal metastases within the same N1 category, despite the clear prognostic differences associated with increasing nodal load. This is consistent with recent evidence that lymphatic dissemination is a distinct biological behavior that is characterized by tumor clones that are more aggressive and capable of vascular and peritoneal invasion. Our lymph node-negative group had a median OS of 42 months, while the node-positive group had a median OS of 26 months. Similar survival differentials have been documented by Hasegawa et al. (2018), who reported a five-year survival of 74% for node-negative versus 41% for node-positive patients. With a significant log-rank p-value (0.003) and a distinct separation between the curves, our Kaplan-Meier analysis confirmed this survival disadvantage [17,18].

Limitations

Several limitations should be acknowledged. First, the retrospective nature of the study introduces potential selection and information biases. The number of lymph nodes retrieved varied between patients, possibly affecting the true detection rate of nodal metastasis. Second, molecular and genetic profiling (e.g., BRCA1/2, p53 mutations) was not available, preventing correlation of nodal status with tumor genomics. Third, follow-up duration was limited to five years, which may underestimate late recurrences. Lastly, the study was conducted at a single tertiary-care center, which may restrict generalizability to the broader population.

## Conclusions

It is concluded that lymph node involvement serves as a crucial prognostic determinant in patients with epithelial ovarian carcinoma. The presence of nodal metastasis was significantly associated with advanced FIGO stage, higher tumor grade, and elevated serum CA-125 levels, indicating a more aggressive tumor biology. Patients with positive lymph node status exhibited substantially lower OS and DFS compared to those without nodal involvement. Furthermore, lymph node metastasis remained an independent predictor of poor prognosis on multivariate analysis, even after adjusting for other clinicopathological factors. This underscores that lymphatic spread is not merely a staging parameter but a marker of disease aggressiveness and potential for early recurrence.
